# Application of laparoscopic modified Bacon operation in patients with low rectal cancer and analysis of the changes in anal function: A retrospective single-center study

**DOI:** 10.3389/fonc.2023.1087642

**Published:** 2023-01-31

**Authors:** Wei Lu, Shujuan Huang, Hui Ye, Shang Xiang, Xiangsheng Zeng

**Affiliations:** ^1^ Department of Colorectal and Anal Surgery, Jingzhou Hospital Affiliated to Yangtze University, Jingzhou, China; ^2^ Department of Respiratory and Critical Care Medicine, Jingzhou Hospital Affiliated to Yangtze University, Jingzhou, China

**Keywords:** Bacon, Dixon, anorectal manometry, surgery, anastomotic leakage, rectal cancer

## Abstract

**Purpose:**

To investigate the value of modified Bacon operation in patients with low rectal cancer.

**Methods:**

Retrospective analysis of 60 patients treated with laparoscopic surgery for low rectal cancer in the Department of Colorectal and Anal Surgery, Jingzhou Hospital affiliated to Yangtze University, from 2019 to 2022, divided into observation and control groups based on the method of the operation (laparoscopic modified Bacon operation group and laparoscopic Dixon operation with prophylactic ileostomy group). We compared the variations between the two groups.

**Results:**

The length of the abdominal surgical incision was shorter in the observation group than in the control group(P<0.05). In the observation group, the length of hospital stay after the first operation was shorter(P<0.05), the both operations time and the second intraoperative bleeding were less(P<0.05), the DET score at one week after the first operation and the VAS after both operations were fewer than in the control group(P<0.05), the postoperative rate of ischemic necrosis of the exposed bowel was higher(P<0.05), and the anal function was poorer in the short term after the second operation compared with the control group(P<0.05), but there was no significant difference between the anal function at 6 months after the second operation compared with the control group(P>0.05).12 months after the second operation, the anal function has recovered to the preoperative level in the observation group(P>0.05).

**Conclusion:**

The laparoscopic modified Bacon operation has smaller abdominal wounds, which reduces postoperative pain; it does not require the use of staplers, which reduces the patient’s financial burden; no postoperative anastomotic leakage occurs, and a more satisfactory anal function can be obtained.

## 1 Introduction

Low rectal cancer is typically treated with Miles surgery to achieve total tumor removal and decrease the recurrence rate. This operation necessitates the removal of the patient’s anus and creation of a permanent enterostomy in the abdominal wall, which has significant physical and mental effects on the patient. Consequently, surgeons have endeavored to preserve the maximum amount of anal function possible. With the advancement of surgical procedures and newer treatment concepts (1–4), the rate of anal preservation in patients with low rectal cancer has increased significantly. Low anterior rectal resection (LAR or Dixon) is the most common anus-preserving operation performed today, but there is a danger of anastomotic leakage. Even with the transition from manual anastomosis to double stapler operation, improved preoperative assessment, and improved postoperative treatment, the incidence of anastomotic leakage documented in the literature has not changed significantly. morbidity ranges from 2.8% to 30%, and mortality from 2% to 16.4% (1, 5, 6).The modified Bacon operation does not necessitate an ostomy and carries no danger of anastomotic leakage; however, some studies (7, 8) indicate that patients have poor postoperative bowel control; therefore, this operation is performed less frequently by some surgeons. In this study, we analyzed patients who underwent laparoscopic modified Bacon operation at Jingzhou Hospital affiliated to Yangtze University in recent years, comparing the changes in anal function before and after surgery with those who underwent laparoscopic low anterior rectal resection (LAR or Dixon) with a prophylactic colostomy. This study provides clinical evidence for selecting a better anus-preserving operation for patients with low rectal cancer.

## 2 Information and methods

### 2.1 General information

From 2019 to 2022, the data of 60 patients who underwent laparoscopic surgery for low rectal cancer at the Department of Colorectal and Anal Surgery, Jingzhou Hospital affiliated to Yangtze University, were analyzed retrospectively. There were 36 males and 24 females; their ages ranged from 36 to 82 years, with a mean of 60.62 years; their disease duration ranged from 3 to 20 months, with a mean of 10.26 months; and their BMI ranged from 17.07 to 27.81, with a mean of 22.56. The distance between the lower margin of the tumor and the anal margin ranged from 2.5 cm to 4.5 cm, with a mean of 3.61cm. According to the eighth edition of tumor staging published by the American Joint Committee on Cancer (AJCC), there were 23 patients in Phase I, 29 in Phase II, and 8 in Phase III. According to the surgical method, the patients were divided into two groups: the control group (laparoscopic Dixon operation with prophylactic ileostomy group) and the observation group (laparoscopic modified Bacon operation group). Before the operation, all patients signed an informed consent form.

### 2.2 Case inclusion and exclusion criteria

#### 2.2.1 Inclusion criteria

(1) pathological diagnosis of malignant rectal tumor; (2) tumor stage I-III according to the American Joint Committee on Cancer (AJCC) 8th edition; (3) the tumor was ≤5 cm from the anal margin; (4) preoperative anal canal manometry was normal;(5) For patients undergoing laparoscopic Dixon surgery, a concomitant prophylactic enterostomy was required; (6) patients received continuous postoperative follow-up at Jingzhou Hospital affiliated to Yangtze University; (7) complete clinical data were available.

#### 2.2.2 Exclusion criteria

(1) Preoperative functional fecal incontinence; (2) Localized invasion of the external sphincter and levator ani muscle by the tumor or with distant metastases from the tumor; (3) Combination of serious underlying diseases such as heart, brain, liver, lung, kidney and hematopoietic system; (4) Conditions such as acute bowel obstruction or bowel perforation due to tumor necessitating emergency surgery; (5) Severe obesity or severe malnutrition; (6) Concurrent multiple primary cancer, recurrent low rectal cancer; (7) Familial adenomatous polyposis; (8) Need for combined organ resection; (9) Combined immune or inflammatory diseases such as autoimmune diseases, inflammatory bowel disease; (10) Mental retardation, neuropsychiatric disorders; (11) Mid-term withdrawal.

### 2.3 Surgical methods

#### 2.3.1 Laparoscopic Dixon operation with prophylactic ileostomy group

**Figure 1:**
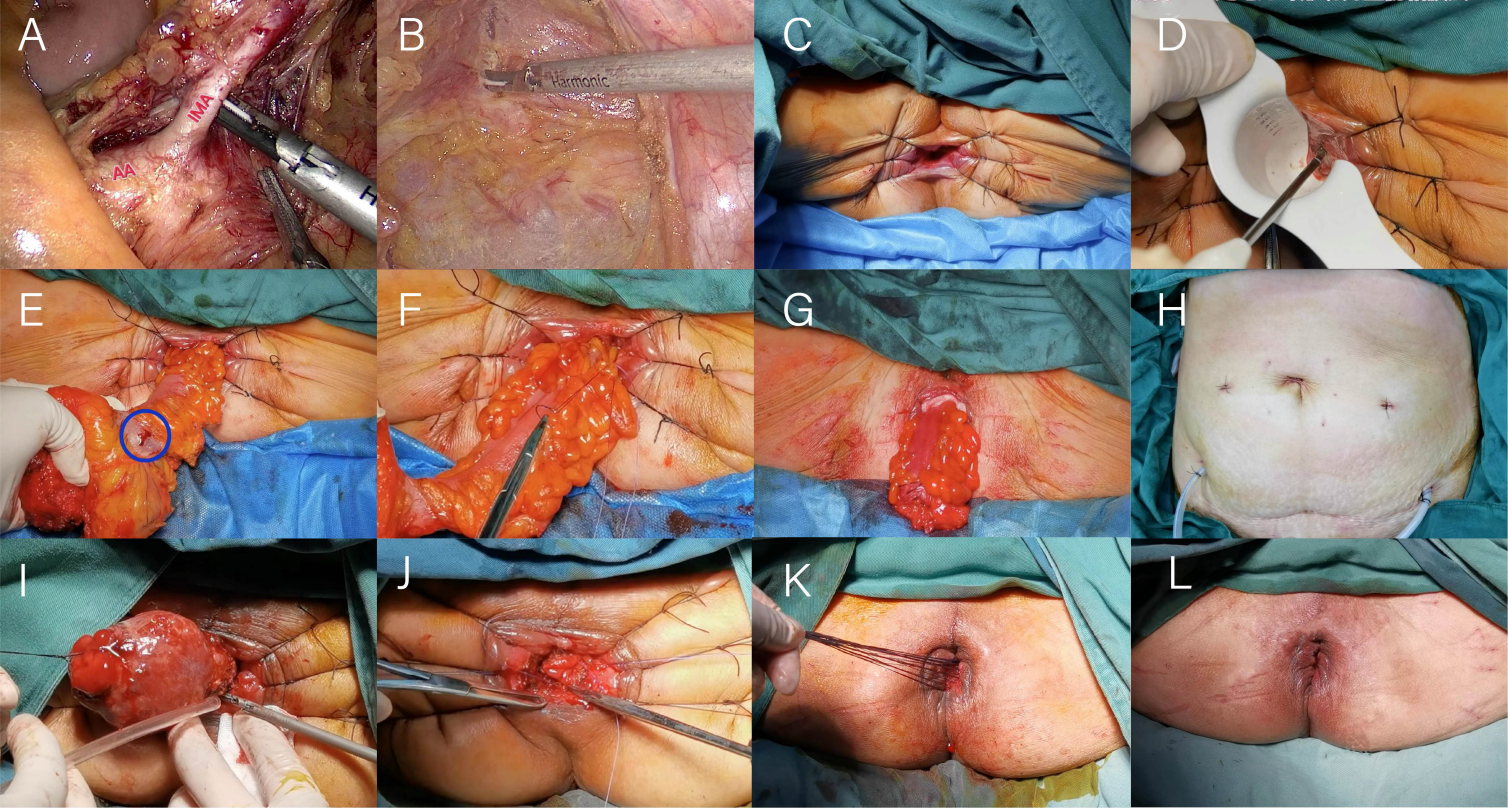
Illustration of the operation of laparoscopic modified Bacon surgery. **(A)** Lymph nodes around the inferior mesenteric artery are cleared. **(B)** The distal end of the rectum is dissociated to the level of the interspace between internal and external sphincters of anal canal. **(C)** Exposure of the surgical field. **(D)** Incision of the whole rectum. **(E)** Pull out the bowel tube and check the blood supply. **(F)** The inner anal colon and the levator ani muscle were fixed together. **(G)** Perineal appearance after resection of tumor bowel. **(H)** Two pelvic drainage tubes were placed. **(I)** Excision of excess colon. **(J)** The sigmoid colon was anastomosed to the rectal stump. **(K)** Unnecessary sutures are cut. **(L)** Perineal appearance after the second operation.

The first operation: Adequate bowel preparation was performed before surgery. The patient is placed in a lithotomy position, routinely disinfected and towelled. A 10 mm diameter observation hole is made 0.5 cm above the umbilicus to create a pneumoperitoneum, and 10 mm and 5 mm trocars are placed in the external 1/3 of the line between the right and left anterior superior iliac spines and the umbilicus, respectively. The retroperitoneum is opened with an ultrasound knife at the sigmoid mesenteric and retroperitoneal reflexes, and the Toldt’s fascia space is entered. Follow the vessel’s course to the inferior mesenteric artery (IMA) root and expose the IMA’s root. The perivascular fat and lymph nodes are cleared (**Figure 1A**). Depending on the situation, the IMA and the inferior mesenteric vein (IMV) are ligated at the root of the IMA or at the preserved LCA, the rectal mesentery is sharply freed along the retrorectal space, and the anterior rectal space is freed along Denonvilliers fascia, the cancerous rectal segment is cut with an intraluminal cutter at least 2 cm from the lower edge of the tumor, the sigmoid colon is cut at about 10-15cm from the upper edge of the tumor, the cancerous bowel segment is removed, and the rectal and sigmoid ends are anastomosed. An anal drainage tube was placed about 10 cm above the anastomosis and fixed to the perianal skin with sutures. Avoid perforation of the rectum and injury to blood vessels, ureters and adjacent organs. The anastomosis is checked for tension. Finally, a prophylactic ileostomy is performed. The terminal ileum (approximately 20 cm from the ileocecum) is lifted with non-invasive forceps by the assistant under direct laparoscopic vision; the intestinal wall is intermittently sutured to the skin around the stoma with 2-0 absorbable thread; the drainage tube is placed, and the incision for the extraction of the specimen and the Trocar holes are sutured; the stoma is completed with an electric knife incision of the intestinal canal. The second operation: the ileum was freed from the surrounding tissues, and the intestinal stoma tube was removed. The small intestine distal and proximal to the stoma was laterally anastomosed with a linear cutting suture, the myoplasmic layer was reinforced with absorbable sutures, and the abdomen was closed following the placement of a drainage tube.

#### 2.3.2 Laparoscopic modified Bacon operation group

The first operation: the intra-abdominal operation, is identical to the laparoscopic Dixon operation. The distal end of the rectum is dissociated to the level of the interspace between internal and external sphincters of anal canal (**Figure 1B**). During the perineal operation, the anus should be fully dilated, the skin of the anal verge should be pulled and fixed in all directions using at least 6 mousse threads to expose the surgical field (**Figure 1C**) entirely, the whole rectal wall should be incised by an ultrasound knife and an electric knife through the anus under direct vision with the aid of a purse-string anoscope (**Figure 1D**); the rectum, tumor and sigmoid colon should be dragged out, ensuring that the anal verge should be more than 15cm from the upper edge of the tumor, check the blood supply of the intestinal canal (**Figure 1E**), the inner anal colon and the levator ani muscle were fixed together to prevent retraction or prolapse of the bowel by intermittent suture with absorbable thread, and disconnect the canal at a distance of more than 10 cm from the proximal end of the tumor (**Figures 1F, G**).Two pelvic drainage tubes were placed (**Figure 1H**). The second operation: the anal canal was gradually separated from the sigmoid colonic adhesions with an electric knife to expose the dentate line and the stump of the rectum from the previous operation. The mesenteric vessels of the sigmoid colon were sutured, the distal end of the mesentery was disconnected by ultrasonic knife, and the sigmoid colon was gradually cut off with the ultrasonic knife after nudging the intestinal wall at the proposed dissection with the electric knife and the excess sigmoid colon specimen was removed (**Figure 1I**). Anastomosis of the proximal sigmoid colon to the rectal stump by interrupted whole layer suture with 3-0 absorbable threads (**Figure 1J**).Unnecessary sutures are cut (**Figure 1K**) and the surgical area is disinfected and the operation is completed (**Figure1L**).

### 2.4 Post-operative management

In both groups, routine anti-infection, nutritional support and symptomatic treatment were given post-operatively, and the abdominal sutures were removed about 1 week after surgery. The control group was given daily stoma care, encouraged to get out of bed early and to eat and drink as early as possible, and was discharged 6-12 weeks later for surgery of stoma closure. In the observation group, patients were in bed after surgery, and the sacrococcygeal section was raised with a soft pad to make the external intestinal tube hang down naturally. The color of the external bowel tube was monitored and flushed with dilute iodine daily, and keep the area dry and clean, and external bowel resection was performed about 3 weeks after surgery as appropriate. All patients in both groups were instructed to perform anal lifting exercises after the second operation.

### 2.5 Observation indicators and follow-up

Patients were followed up through a combination of outpatient visits and telephone calls. The follow-up period was from the discharge of the first operation to 1 year after the discharge of the second operation. Patients’ basic information [gender, age, tumor stage, time of operations(1st, 2nd), intraoperative bleeding (1st, 2nd), abdominal wound size (total length), hospital stay after operations(1st, 2nd), interval between two operations, degree of wound pain on the first day after two operations (VAS visual analogue pain scale), observation of postoperative anastomotic leakage, peristomal dermatitis or perianal dermatitis (DET score), hemorrhage, ischaemic necrosis, postoperative low anterior resection syndrome (LARS score) at 3, 6 and 12 months after stoma closure or drag-out segment of bowel resection. Wexner scores and anorectal manometries were performed at preoperation and 1, 2, 3, 6 and 12 months postoperatively to monitor the patient’s recovery of anal function.

The DET score, which consists of three domains, Discoloration (D), Erosion (E) and Tissue overgrowth (T), is obtained by adding the score of the affected area and the severity score for each of the three domains to give a score ranging from 0 to 15. 0 means that the peristoma skin is healthy, and as the score increases, the severity of the problem with the peristoma skin increases.

Pain is scored on a Visual Analogue Scale (VAS) of 0-10 according to the severity of the pain.

The low anterior resection syndrome score, which addresses the five most important clinical signs of low anterior resection syndrome, such as urgency, frequency, clustering, incontinence for flatus and incontinence for liquid stool, the related questions were designed and scored. Based on the total score, the low anterior incision syndrome was classified as no LARS (0-20 points), minor LARS (21-29 points) and major LARS (30-42 points). The higher the score, the worse the anal function.

Wexner score for anal incontinence scored in terms of solid, fluid and gas control, frequency of pad use and degree of lifestyle change, with 0 being normal and 20 being incontinent.

Anal canal rectal pressure measurement by direct manometry (solid catheter), the maximum value of anal canal pressure measured in a calm state (anal canal resting pressure) with a standard reference value of 50 ~ 70 mmHg and the maximum value of anal canal pressure during continuous active anal contraction (anal canal maximum squeeze pressure) with a standard reference value of 110 ~ 140 mmHg.

### 2.5 Statistical analysis

Data analysis was processed using SPSS 25.0 statistical software. The data results of measurement data were expressed as x̄ ± s, and the comparison between measurement data was analyzed by t-test or One-Way ANOVA. Chi-square test or Fisher’s exact test for comparison between two groups for count data. p<0.05 means the difference is statistically significant.

## 3 Results

**Figure 2:**
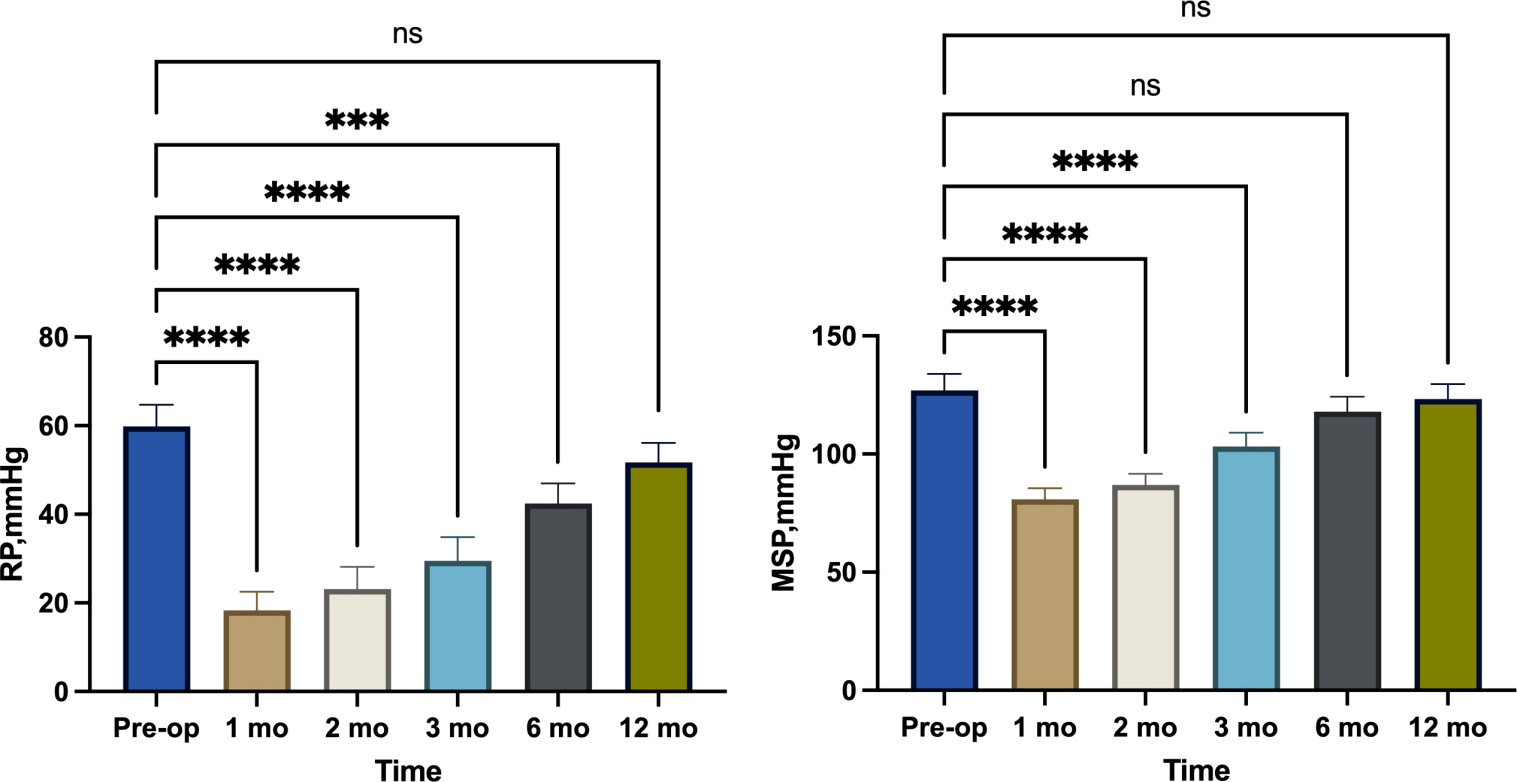
Comparison of resting pressure (RP) and maximum squeeze pressure (MSP) changes in observation group. ***, p<0.001; ****, p<0.0001 ns (no significance), p>0.05.

From January 2019 to August 2022, 60 surgical patients suitable for inclusion in this study were collected through inclusion and exclusion criteria. 28 patients were assigned to the observation group, whereas 32 were assigned to the control group. 36 of the 60 patients with low rectal cancer were men, whereas 24 were women (male-to-female ratio = 3:2. Patients had a mean age of 60.62 years and a mean BMI of 22.56. The average distance between the tumor’s lower margin and the anal margin was 3.61 cm. 23 individuals had tumors at stage 1, 29 patients at stage 2, and 8 patients at stage 3. Separate comparisons of the basic clinical data of the patients in the two groups revealed that the differences were not statistically significant (P > 0.05) (**Table 1**). The abdominal surgical incision was shorter in the observation group compared to the control group(P<0.05). In the observation group, the first intraoperative bleeding volume were not significantly different from those in the control group (P > 0.05), and the both operations time and second intraoperative bleeding volume were significantly less than those in the control group (P < 0.05), but the length of their first postoperative hospital stay was shorter than that of the control group (P < 0.05), and the length of their second postoperative hospital stay was not significantly different from that of the control group (P > 0.05). There was no statistical difference in the results of the second preoperative blood biochemical examination (leukocyte count, neutrophil count and albumin concentration) between the two groups (**Table 2**), and in the observation group and the control group, 2 cases and 1 case, respectively, had leukocyte count exceeding 10*10^9^/L, and were mainly increased neutrophil count, but no pelvic abscess was found. The postoperative related complications were compared, with the occurrence of hemorrhage, anastomotic leakage and anal stenosis, there were no statistical significant differences in the number of cases compared (P > 0.05); there was a statistically significant difference in the number of cases of ischaemic necrosis between the two groups(P < 0.05), and the DET score at one week after the first operation and the VAS score at first day after both operations were significantly less in the observation group than in the control group (P < 0.05)(**Table 3**). At 1, 2, and 3 months postoperatively, the observation group’s Wexner score and anal canal resting pressure were substantially lower than the control group’s (P<0.05). In contrast, there was no statistically significant difference between the two groups in terms of maximum squeeze pressure at 3 months postoperatively (P>0.05). There was no statistically significant difference in Wexner score, anal canal resting pressure and maximum anal canal squeeze pressure between the two groups at preoperation and 6 months postoperatively (P > 0.05) (**Table 4**). 12 months after the second operation, the anal function has recovered to the preoperative level in the observation group(P>0.05) (**Figure 2**).There was a statistically significant difference in LARS scores between the two groups at 3 months and 6 months postoperatively (P < 0.05), and no statistically significant difference in LARS scores at 12 months after operation when compared (P > 0.05) (**Table 5**).

**Table 1:** Patient characteristics.

Characteristics	Group		χ^2^	*t*	* P *
	Bacon (N = 28)	Dixon (N = 32)			
Sex, male/female	17/11	19/13	0.01		0.92
Age, years	61.50 ± 8.85	59.84 ± 11.13		0.63	0.11
BMI, kg/m^2^	22.91 ± 1.55	22.21 ± 1.64		-0.31	0.22
Distance from tumor to anal margin, cm	3.32 ± 0.46	3.86 ± 0.46		-4.12	0.12
Stage			0.94		0.63
I	10	13			
II	13	16			
III	5	3			

**Table 2:** Data on patient hospitalizations and surgeries.

Intraoperative and postoperative data	Group		*t*	*P*
	Bacon (N = 28)	Dixon (N = 32)		
Length of abdominal incision,cm	4.38 ± 0.42	16.31 ± 1.45	-42.09	0.00
Interval between two operations,day	24.79 ± 3.81	50.94 ± 6.24	-19.24	0.09
Length of hospital stay after operation,day				
The first operation	10.96 ± 1.50	12.22 ± 1.18	-3.61	0.04
The second operation	7.61 ± 1.37	10.38 ± 1.21	-8.31	0.63
Time required for operation of the two groups,min				
The first operation time	222.46 ± 29.07	274.69 ± 24.46	-7.56	0.03
The second operation time	63.50 ± 10.77	112.38 ± 26.63	-9.08	0.01
Intraoperative bleeding volume in the two groups,ml				
The first bleeding volume	43.75 ± 36.28	35.63 ± 34.02	0.90	0.63
The second bleeding volume	7.86 ± 4.13	20.63 ± 10.83	-5.87	0.00
Blood biochemical indexes before the second operation				
Leukocyte count, *10^9/^L	8.25 ± 1.65	7.61 ± 1.46	1.58	0.54
Neutrophil count, *10^9/^L	4.81 ± 1.67	4.67 ± 1.40	0.36	0.30
Albumin concentration, g/L	40.11 ± 2.43	41.51 ± 2.84	-1.73	0.28

**Table 3:** Comparison of postoperative complications.

Complications	Group		χ^2^	*t*	*P*
	Bacon (N = 28,%)	Dixon (N = 32,%)			
Hemorrhage	1(3.57)	3(9.38)	0.81		0.37
Ischemic necrosis	4(14.29)	0(0.00)	4.90		0.03
Anastomotic Leakage	0(0.00)	1(3.33)	0.89		0.35
Anal stenosis	1(3.57)	2(6.3)	0.23		0.64
DET score(1 week after the 1^st^ operation)	1.67 ± 0.54	4.31 ± 0.82		-14.40	0.04
VAS score (1 day after both operations)					
The first operation	0.82 ± 0.61	4.56 ± 1.05		-16.60	0.00
The second operation	2.29 ± 0.54	4.66 ± 0.79		-13.44	0.03

**Table 4:** Changes in anal function in patients without operation and after the second operation.

Anal function	Group		*t*	*P*
	Bacon (N = 28)	Dixon (N = 32)		
Wexner score				
Preoperation	1.14 ± 0.36	1.06 ± 0.50	0.70	0.59
1 month after operation	16.54 ± 1.11	11.38 ± 1.88	12.73	0.02
2 month after operation	12.93 ± 2.28	9.00 ± 1.52	7.94	0.01
3 months after operation	9.75 ± 2.32	7.38 ± 1.45	4.82	0.01
6 months after operation	6.75 ± 1.67	4.72 ± 1.37	5.17	0.16
12 months after operation	3.32 ± 0.98	2.94 ± 0.88	1.60	0.42
Anorectal manometry,mmHg				
Preoperation				
Resting pressure	59.86 ± 4.87	60.09 ± 5.37	-0.18	0.32
Maximal squeeze pressure	126.89 ± 7.09	128.28 ± 6.14	-8.12	0.31
1 month after operation				
Resting pressure	18.36 ± 4.16	34.56 ± 2.99	-17.48	0.03
Maximal squeeze pressure	80.86 ± 4.74	93.63 ± 7.42	-7.81	0.01
2 months after operation				
Resting pressure	23.14 ± 4.98	39.47 ± 3.26	-15.18	0.02
Maximal squeeze pressure	86.90 ± 4.79	99.47 ± 7.16	-7.87	0.01
3 months after operation				
Resting pressure	29.05 ± 5.32	44.03 ± 3.18	-13.03	0.00
Maximal squeeze pressure	103.07 ± 5.86	107.94 ± 7.22	2.84	0.10
6 months after operation				
Resting pressure	42.43 ± 4.67	52.47 ± 3.75	-9.35	0.27
Maximal squeeze pressure	117.93 ± 6.36	120.25 ± 5.58	-1.51	0.92
12 months after operation				
Resting pressure	51.75 ± 4.38	56.00 ± 4.70	-3.61	0.68
Maximal squeeze pressure	123.29 ± 6.32	124.89 ± 6.27	-0.98	0.93

**Table 5:** Postoperative recovery of low anterior resection syndrome in two groups.

LARS score	Group		χ^2^	*P*
	Bacon (N = 28)	Dixon (N = 32)		
**3 months after operation**			8.08	0.02
No LARS	2	6		
Minor LARS	10	19		
Major LARS	16	7		
**6 months after operation**			6.68	0.04
No LARS	6	13		
Minor LARS	12	16		
Major LARS	10	3		
**12 months after operation**			0.61	0.74
No LARS	14	18		
Minor LARS	12	13		
Major LARS	2	1		

## 4 Discussion

Globally, there are approximately 1.9 million new cases and 900,000 deaths from colorectal cancer each year, making it the third most common cancer disease and the second leading cause of cancer death, according to the International Agency for Research on Cancer in 2020 (9).

At present, the mainstay of anal preservation surgeries for low rectal cancer are low anterior resection (LAR or Dixon), intersphincteric resection (ISR) and colo-anal anastomosis (Parks). However, all of them carry the risk of anastomotic leakage or require a prophylactic stoma, which may further increase the physical and psychological burden of the patients.

In order to avoid the risk of anastomotic leakage, the concept of a drag-out rectal resection was introduced by the Viennese surgeon Julius von Hochenegg in 1887 under the name Durchzieh method (10), which was later refined by William Wayne Babcock and Harry E. Bacon, who introduced the Bacon method in 1945 (11). Although this operation could completely remove the rectal tumor, the removal of the entire rectum caused damage to the anorectal ring, and the patient’s postoperative anal function was poor (12, 13). As a result, it was conducted infrequently in the clinic at the time. Professor Zhou Xigeng changed the Bacon operation four times between 1954 and 1991 (14). With the rapid development of laparoscopic operations and minimally invasive treatment approaches, the modified Bacon operation has gradually transitioned from the original open operation to a laparoscopic operation in which the specimen is extracted through the anus. This operation permits a greater exposure of the anatomical level of the deep pelvic tissues adjacent to the anus, facilitates intraoperative protection of the pelvic nerves, and decreases the incidence of postoperative pelvic infection. This operation not only satisfies the need for anal preservation in patients with low rectal cancer but also eliminates the need for an adjuvant abdominal incision and prophylactic stoma and is more favorable to enhancing the postoperative quality of life for patients.

In this study, the tumor specimen of the control group had to be removed *via* a separate abdominal incision, and in addition to the prophylactic stoma, the postoperative abdominal wound was larger, and the patient’s suffering was more severe than in the observation group. The average stoma closure time was 50.94 days, mainly to enable the anastomosis to heal well and reduce the risk of anastomotic leakage. During this time, the patient required more stoma bags and stoma care products, which increased their financial burden. In addition, some stoma-related complications can also occur because of the presence of the stoma.32 patients in the control group developed varying degrees of dermatitis in the early postoperative period as a result of the stoma being almost at the same level as the skin, the stoma bag chassis not fitting the skin, the high likelihood of feces coming in contact with the skin, and the mechanical damage caused by the pulling of the sump backing on the skin during stoma bag changes (15). In addition, when the patient moves, the stoma tends to rub against the cut edge of the stoma bag chassis, which increases the risk of intestinal oedema and bleeding. In the observation group, the external bowel tube was longer and parallel to the torso, and most patients were bedridden following the initial operation; hence, the feces were less likely to come into touch with the surrounding skin, and dermatitis was less. In this study, four cases of ischaemic necrosis (there were 2 cases of partial necrosis of the distal end of the exposed colon, and 2 cases of necrosis below the anorectal ring plane, without adverse consequences.) occurred in the observation group, which was partly related to surgical techniques, primarily due to the inadequate dilation of the anal canal resulting in the trapping of the anorectal ring, closure of the marginal vessels during suture fixation. In addition, any situation with pressure on the external bowel has a high chance of causing ischaemic necrosis; therefore, modified Bacon operation may not be suitable for patients with a short length of sigmoid colon and mesentery, excessive obesity, preoperative anal stenosis and sphincter tension is high, and certainly not for patients with large tumors, abdominal organ prolapse into the pelvis minor, or preoperative anal incontinence. Postoperative management also requires that the patient be advised to elevate the buttocks with a cushion to prevent ischaemic necrosis of the bowel due to compression.

**Figure 3:**
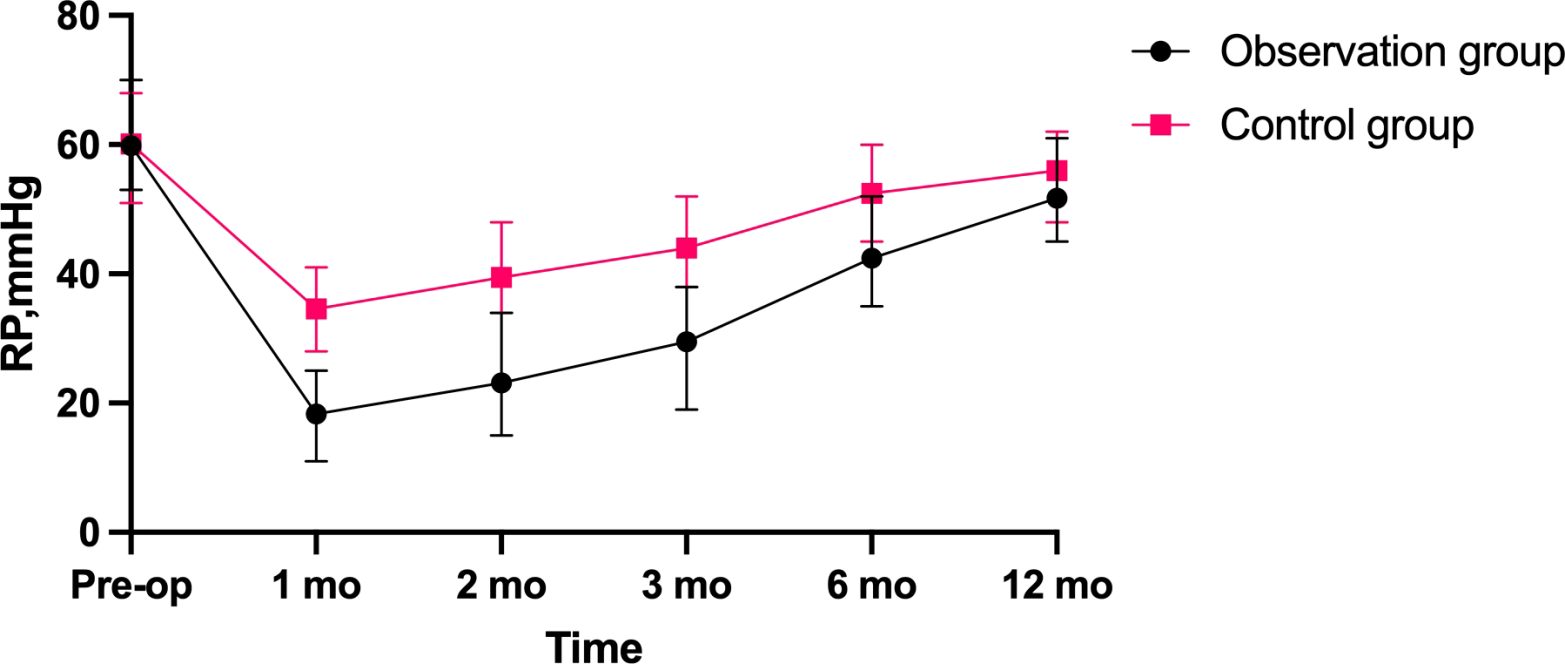
Comparison of resting pressure (RP) changes in anal canal between two groups.

**Figure 4:**
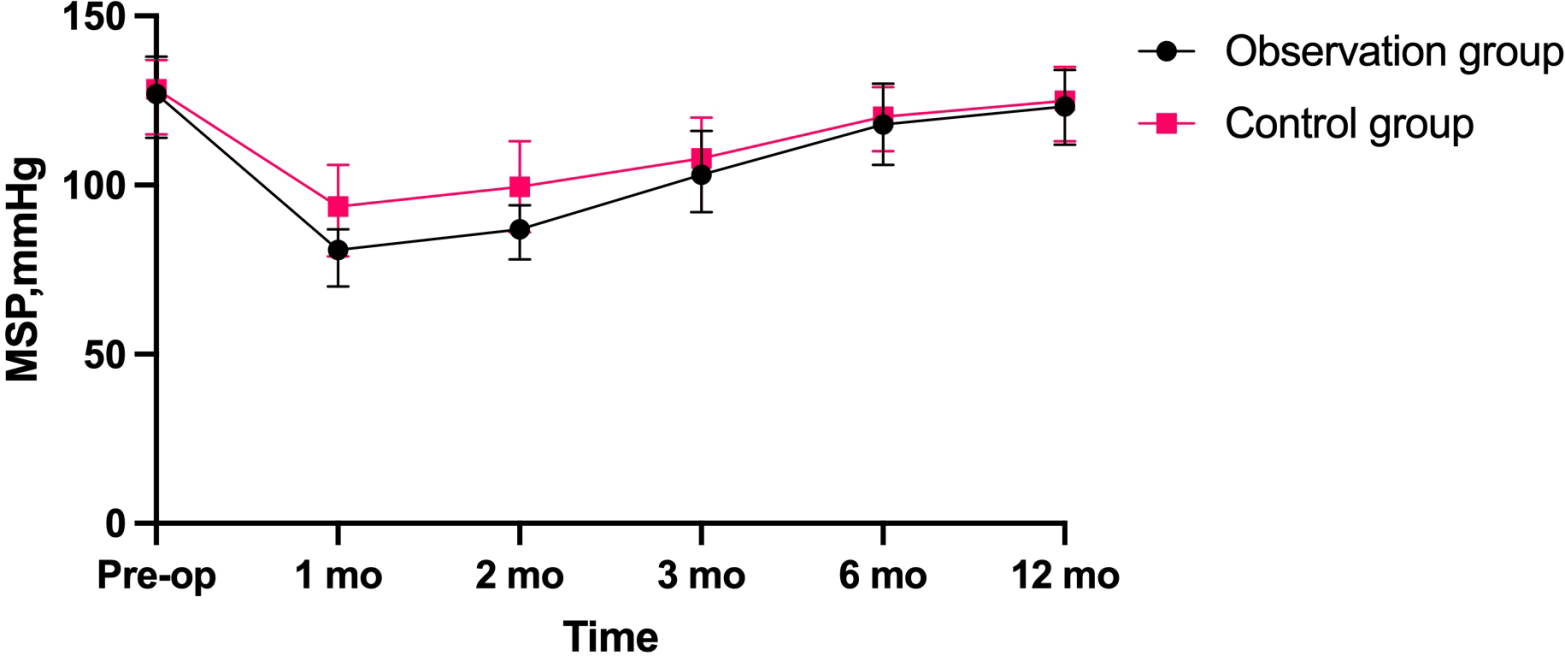
Comparison of maximum squeeze pressure (MSP) changes in anal canal between two groups.

As a result of the short abdominal incision, the absence of an abdominal stoma, and the fact that larger tumor specimens did not need to be removed by re-opening the abdomen, patients in the observation group experienced less postoperative pain than those in the control group. The second postoperative pain was still less severe than that of the control group, mostly because the majority of procedures were performed above the dentate line, which is innervated by autonomic nerves. The VAS was statistically higher in the observation group after the second operation compared to the first, which may be related to the irritation of the external bowel tube and dressing that caused external hemorrhoid oedema; consequently, some patients with external hemorrhoid oedema require active treatment to reduce oedema. In the control group, the long interval between the two operations necessitated the use of stoma bags and stoma care products, which added to the patient’s financial burden, whereas in the observation group, the average interval between the two operations was 24.79 days and no stoma pouches were required, which reduced the patients’ psychological and financial stress. The both operations time of the control group was significantly longer than that of the observation group because the surgeons need to open and close the abdomens and make ostomies in the first operation, and in the second operation, they must separate the adherent intestines to facilitate the subsequent intestinal anastomosis due to the irritation of the first operation of the control group, which increases the likelihood of accidental vessel injury and consequently increases the amount of bleeding. In addition, in the control group, some patients also needed to deal with parastostomal hernia, which extended the operation time. The second operation of observation group can be finished under spinal anesthesia without re-entering the abdomen, and the average duration is 63.50 minutes, which is less than that of the control group. Additionally, there is less bleeding and no need for consumables such as staplers, which decreases the patient’s financial burden. In the observation group, the Wexner score was significantly higher than that of the control group at 1, 2 and 3 months after second operation, and the resting pressure of the anal canal was lower than that of the control group at 1, 2 and 3 months after the second operation (**Figure 3**), the difference was statistically significant, probably because the modified Bacon operation disrupted the pratial internal sphincter and had poorer short-term anal control, but there was no significant difference in Wexner scores and resting pressure between the two groups at 6 months after the second operation compared with that of control group. In this study, we found a significant difference in the maximum squeeze pressure of the anal canal between the two groups at 1 and 2 months after the second operation, with the observation group significantly lower than the control group (**Figure 4**), which may be caused by the surgery itself, such as intraoperative excessive anal dilatation result in the external sphincter injury, or a period of intestinal tube dragging out, causing spasmodic contraction of the anal sphincter, resulting in relaxation of the anal sphincter and a decrease in muscle strength, we instructed the patients to do more postoperative exercises to lift the anus, and the maximum squeeze pressure was measured at 3, 6 and 12 months after the second operation, and there was no significant difference between the observation group and the control group. Moreover, the analysis found that 12 months after the second operation, the anal function has recovered to the preoperative level in the observation group(P>0.05) (**Figure 2**), which shows that modified Bacon operation can achieve a satisfactory anal dynamic result.

Low anastomosis is one of the most prominent risk factors for LARS (16). The height of the anastomosis has a direct effect on the occurrence of LARS. Patients who have undergone low anterior resection and other ultra-low anastomosis operations for rectal cancer may develop varying degrees of bowel dysfunction, including urgency, frequency, impaired evacuation, and fecal incontinence (17). Low Anterior Resection Syndrome describes a group of syndromes (LARS).The LARS-specific scale designed by Emmertsend et al. (18, 19) addresses the five most significant clinical symptoms of LARS, with the items being “urgency, frequency, clustering, incontinence for flatus,” and “incontinence for liquid stool.” Accordingly, each item is granted a score based on its association with quality of life impacts. The scores ranged from 0 to 42, with 0-20 (no LARS), 21-29 (minor LARS), and 30-42 (major LARS). In this study, the LARS scores of the observation group at 3 and 6 months postoperatively were significantly different from those of the control group. The impact on the quality of life of the observation group was greater in the short term, with 16 patients in Major LARS at 3 months postoperatively and 3 patients having up to 15 bowel movements per day, which had a more significant negative impact on working life, but these conditions improved significantly 12 months postoperatively.

## 5 Conclusion

The laparoscopic modified Bacon operation has no incision and no stoma in the abdomen, thereby avoiding complications and other effects caused by incision and stoma; there is no anastomosis in the first stage of the operation, so there is no need to worry about anastomotic leakage; therefore, the modified Bacon operation is suitable for older patients or those with high risk factors for anastomotic leakage such as diabetes, chronic obstructive pulmonary disease, and hypoproteinemia; it can improve the rate of anal preservation, and can be used as a remedy for the failure of other anal preservation operation, and is also suitable for cases where it is challenging to use staplers or other instruments to perform anastomosis or where the need for a prophylactic stoma is estimated after anastomosis. In addition, rectal benign tumors or other rectal tumors that cannot be locally resected (e.g., large villous adenoma of the rectum, rectal gastrointestinal stromal tumor) can be treated by modified Bacon operation. The short-term impact on anal function is significant, but in the long term, the indicators of anal function are not significantly different from those of Dixon. Although there was no statistical difference between the two groups in terms of the interval between the two operations, the interval between the two operations was generally longer in the control group than in the observation group; therefore, the comparison of anal function between the two groups may be subject to error. In summary, the laparoscopic modified Bacon operation is superior to the laparoscopic Dixon operation with prophylactic ileostomy in the treatment of patients with low rectal cancer, and it is a preferred method to preserve anus in the treatment of low rectal cancer.

## Data availability statement

The original contributions presented in the study are included in the article/supplementary material. Further inquiries can be directed to the corresponding author.

## Ethics statement

The studies involving human participants were reviewed and approved by Ethics Committee of Jingzhou Central Hospital. The patients/participants provided their written informed consent to participate in this study. Written informed consent was obtained from the individual(s) for the publication of any potentially identifiable images or data included in this article.

## Author contributions

Conceptualization, HY, WL, and SH. Methodology, HY. Software, SX. Validation, XZ. Formal analysis, WL and SH. Investigation, WL and SH. Resources, HY. Writing—original draft preparation, WL and SH. Writing—review and editing, HY. Supervision, HY. Project administration, HY. All authors contributed to the article and approved the submitted version.
